# How Skill Expertise Shapes the Brain Functional Architecture: An fMRI Study of Visuo-Spatial and Motor Processing in Professional Racing-Car and Naïve Drivers

**DOI:** 10.1371/journal.pone.0077764

**Published:** 2013-10-18

**Authors:** Giulio Bernardi, Emiliano Ricciardi, Lorenzo Sani, Anna Gaglianese, Alessandra Papasogli, Riccardo Ceccarelli, Ferdinando Franzoni, Fabio Galetta, Gino Santoro, Rainer Goebel, Pietro Pietrini

**Affiliations:** 1 Laboratory of Clinical Biochemistry and Molecular Biology, University of Pisa, Pisa, Italy; 2 Clinical Psychology Branch, University of Pisa, AOUP Santa Chiara, Pisa, Italy; 3 MRI Laboratory, Fondazione Regione Toscana/CNR ‘G.Monasterio’, Pisa, Italy; 4 Formula Medicine, Viareggio, Italy; 5 Sport Medicine Unit, Department of Clinical and Sperimental Medicine, University of Pisa, AOUP Santa Chiara, Pisa, Italy; 6 Maastricht Brain Imaging Center, Universiteit Maastricht, Maastricht, Netherlands; Università di Trento, Italy

## Abstract

The present study was designed to investigate the brain functional architecture that subserves visuo-spatial and motor processing in highly skilled individuals. By using functional magnetic resonance imaging (fMRI), we measured brain activity while eleven Formula racing-car drivers and eleven ‘naïve’ volunteers performed a motor reaction and a visuo-spatial task. Tasks were set at a relatively low level of difficulty such to ensure a similar performance in the two groups and thus avoid any potential confounding effects on brain activity due to discrepancies in task execution. The brain functional organization was analyzed in terms of regional brain response, inter-regional interactions and blood oxygen level dependent (BOLD) signal variability. While performance levels were equal in the two groups, as compared to naïve drivers, professional drivers showed a smaller volume recruitment of task-related regions, stronger connections among task-related areas, and an increased information integration as reflected by a higher signal temporal variability. In conclusion, our results demonstrate that, as compared to naïve subjects, the brain functional architecture sustaining visuo-motor processing in professional racing-car drivers, trained to perform at the highest levels under extremely demanding conditions, undergoes both ‘quantitative’ and ‘qualitative’ modifications that are evident even when the brain is engaged in relatively simple, non-demanding tasks. These results provide novel evidence in favor of an increased ‘neural efficiency’ in the brain of highly skilled individuals.

## Introduction

Selected populations of individuals achieve very high levels of skills and performance in fields ranging from arts to sport activities as a consequence of intensive training and, probably, of some genetic predisposition [1]. Brain functional studies have begun to indicate that skill acquisition in different perceptual, motor or cognitive domains may be associated with response modifications, either in extension or magnitude, in task-associated brain areas [2-4]. Specifically, these modifications include both increased or decreased response in task-related regions, which may also be differentially combined, giving rise to distinct patterns of cortical functional reorganization [3]. The expansion of sensory and/or motor topographic representations, such as in the prototypical cases of the auditory and motor cortex of musicians, are well-known examples of practice-related increased responses [5,6]. On the other hand, a reduction in neural response has been observed both in longitudinal studies investigating the effects of cognitive or motor learning [7,8] and in experiments comparing novices and distinct categories of experts and skilled individuals, such as golf players or archers [9-16]. While a decrease in brain response can be interpreted as a sign of enhanced efficiency in regional resource utilization, a number of studies also reported a reallocation of neuronal resources based on both a decreased response in cognitive control areas and an enhanced activity in other brain regions (reviewed in 2,4). For instance, an increased activity within the so called ‘default mode network’ regions [17] has been shown [4]. As the default mode network represents a system of interconnect brain areas that are more active during ‘internal’ rather that ‘external’ tasks (e.g., mind-wandering, memories recollection or envisioning of the future [18,19]), these findings suggest that neural efficiency may be associated with a greater automaticity and a reduced attentive load during task execution [4]. 

Moreover, functional and effective connectivity analyses revealed that these functional changes in brain response may be accompanied also by modifications in the way task-related regions interact, usually with a strengthening of the essential couplings and a pruning of the ‘unnecessary’ ones [20-26]. 

Altogether, these observations support the so called ‘*neural efficiency*’ hypothesis, which postulates a more efficient cortical functioning based on both a reduced utilization of resources and an improvement in information processing, thanks to a better communication between task-related brain areas, in expert/skilled as compared to ‘ordinary’ individuals [15,27-29]. Interestingly, studies exploring age-related functional changes during distinct perceptual and cognitive tasks revealed complementary findings, demonstrating that healthy older individuals often recruit a greater volume of brain cortical areas [30-32] and show a reorganization of associated functional networks [33,34], as compared to younger adults. These modifications are commonly interpreted as an attempt of the brain to compensate for an age-related impairment in neural efficiency [35,36]. Furthermore, older individuals are characterized by a reduction in blood oxygen level-dependent (BOLD) signal temporal variability [37], a measure regarded as a marker of complexity of information integration and functional efficiency [37-40].

While we begin to know the functional changes that accompany practice in specific tasks, to our knowledge no study has explored yet the brain functional correlates associated with exposure to extreme training and competing conditions, like those involved in high speed car-racing. As a matter of fact, this sport discipline exposes to some of the most demanding conditions one can imagine the human brain to endure, requiring exceptional skills in terms of visuo-spatial processing, motor control, decision making and sustained attention. Top-level Formula racing-car drivers ordinarily undergo intensive psychophysical training and are exposed to extreme competitive conditions (e.g., accelerations 0-100 km/h in 1.7 s, top speeds up to 360 km/h, need for high concentration levels and precise sensory-motor control, etc.), which unquestionably represent a huge effort for both their body and brain. In addition, most of them have a history of ‘high-speed activities’ (e.g., go-kart or motor racing) since a very young age, when brain plasticity is at its maximum [41]. 

Thus, the present study was designed to assess the brain functional correlates associated with visuo-spatial and motor processing in a group of elite Formula racing-car drivers. Specifically, we hypothesized that visuo-spatial and motor processing in these highly skilled individuals would be associated with a significantly more efficient use of brain resources in comparison with a matched group of untrained ‘naïve’ drivers. To test this hypothesis, functional Magnetic Resonance Imaging (fMRI) was used to measure brain activity while professional and naïve drivers performed a motor reaction task and a multiple target pursuit (visuo-spatial) task. These tasks required relatively simple perceptual and motor skills in order to avoid any performance differences between the two groups and consequent biases on neural response [42]. To evaluate potential differences in brain functional efficiency between the two groups, we measured both cortical regional brain response and patterns of inter-regional interactions, as well as regional levels of BOLD signal temporal variability.

## Material and Methods

### Subjects

Eleven professional (mean age ± S.D. = 24 ± 4 years) and 11 naïve (28 ± 4 years, p= n.s.) car drivers were studied. All subjects were right-handed healthy males. Professional car drivers were recruited through the Formula Medicine® group (Viareggio, Italy), were actively participating in a professional Formula racing tournament (as Formula One Championship, World Series, Formula 3, etc.) at the time of the study, and had a minimum of four years of expertise in amateur and/or professional racing. All participants received medical, neurological and psychiatric examinations, and laboratory exams, including blood tests and a structural brain MRI scan exam, to rule out history or presence of any disorder that could affect brain function and development. No participant was taking any medication. 

### Ethics Statement

All volunteers gave their written informed consent after the study procedures and risks involved had been explained. The study was conducted under a protocol approved by the University of Pisa Ethical Committee (protocol n. 1616/2003), and was developed in accordance with the Protocol of Helsinki (2008). All participants retained the right to withdraw from the study at any moment.

### Image Acquisition

Functional data were acquired on a GE Signa 1.5 Tesla scanner (General Electric, Milwaukee, WI) using following parameters: repetition time = 2,500 ms, 21 axial-slices, slice thickness = 5 mm, field of view = 24 cm, echo time = 40 ms, flip angle = 90°, image plane resolution = 128 x 128. Subjects were presented with a six run block design study including randomly-alternated motor reaction and visuo-spatial tasks. Every run was constituted by three task blocks (each 60 s duration) alternated with two inter-task intervals (ITI, each 25 s duration). Each time series began and ended with 25 s of no stimuli, similarly to the ITI. For each subject, we obtained 2-3 time series of 112 brain volumes (280 s) for the motor reaction task and 3 time series for the visuo-spatial task. Visual stimuli were presented on a rear projection screen viewed through a mirror (visual field: 25° wide and 20° high). Before the fMRI scanning, subjects assisted to a demonstrative session to become familiar with the task procedure. To enhance compliance and participation in the tasks, the experiment was presented as a competition, in which recorded performances would be used to measure individual abilities and to establish a ranking.

For each subject we also obtained a high-resolution T_1_-weighted spoiled gradient recall image (134 slices, slice thickness = 1 mm, echo time = 3.8 ms, repetition time = 20 ms, flip angle = 15°, field of view = 22 cm) to provide detailed brain anatomy for functional data localization.

### Motor Reaction Task

**Figure 1:**
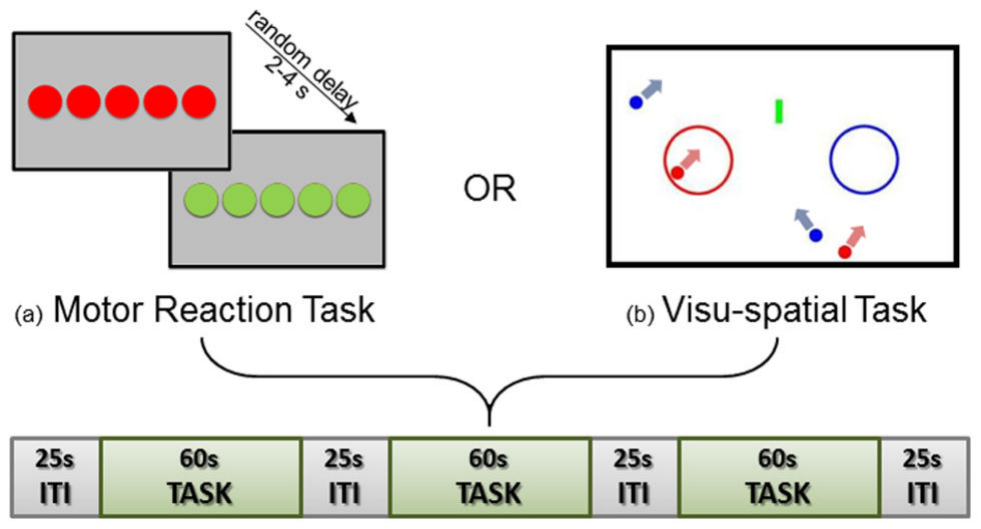
Experimental paradigm. Every run was constituted by three task blocks (each 60 s duration) alternated with two inter-task intervals (ITI, each 25 s duration). During the Motor Reaction task (a), subjects had to press a button as quick as possible when the red light turned green (random delay comprised between 2 and 4 s). During the Visuo-spatial task (b) volunteers were required to press a button when one of the moving balls entered the color-matched circle.

During the motor reaction task, the visual stimulus reproduced a starting grid light on a gray background, and was constituted by five red circles arranged horizontally that turned to green after a random delay comprised between 2 and 4 s (Figure 1a). This condition was repeated ten times for each task-block (total block duration of 60 s; 24 brain volumes per block). Participants were asked to fixate a white static central cross (0.15° x 0.15°) and to use their right thumb to press a response button held in their right hand as rapidly as possible when the starting grid color turned to green. Reaction times, defined as the time from the stimulus onset to the key-button response, were automatically recorded. The intervals between the blocks presented a 25 s static image with the white fixation cross on the gray background. We collected 2-3 time series for each subject of the two experimental groups, with the exception of a professional driver that was excluded for technical problems during image acquisition (thus data were acquired in 11 naïve and 10 professional car drivers). Stimulus presentation was handled by using the software package Presentation (http://www.neurobehavioralsystems.com).

### Visuo-Spatial Task

During this visuo-spatial task, subjects were asked to observe a left-sided red and a right-sided blue circle inside a white ‘billiard table-like’ frame, arranged horizontally on a black background (Figure 1b). Two red and two blue balls moved randomly within the ‘billiard’, and participants had to press a left-hand or a right-hand held button when a ball run inside the color-matched circle. A vertically moving small green ‘barrier’ was also present in the center of the frame to interfere with the movement of the colored balls. All subjects responded using their left thumb for the red circle/target and their right thumb for the blue circle/target. For each subject, correct, wrong and missed answers were automatically recorded. As in the motor reaction task, in each run, three 60 s long task-blocks were separated by 25 s of static billiard frame. Stimulus presentation was handled by using the software Formula Test 2003 (Maxisoft^®^, Massa, Italy).

### Behavioural Data Analysis

Behavioural data analyses were carried out using StatView software 5.0 (SAS Institute Inc.). In the visuo-spatial task, error rates, defined as percent of errors on the total given responses, were taken as an estimation of individual task accuracy. Reaction times collected during the motor reaction task and error rates relative to the visuo-spatial task were used in unpaired two-tailed *t*-tests to search for differences in performance between the two experimental groups. 

### Functional Images Analysis

We used AFNI and SUMA software packages to preprocess, analyze and view functional imaging data (http://afni.nimh.nih.gov/afni; [43]). For the two tasks, all volumes obtained in the respective runs were concatenated and coregistered (*3dvolreg*), temporally aligned (*3dTshift*), and spatially smoothed (FWHM = 6 mm). Individual run data were normalized by calculating the mean intensity value for each voxel during resting baseline timepoints, and by dividing the value within each voxel by this averaged baseline to estimate the percent signal change at each time point.

Multiple regression analyses were performed to independently identify brain regions significantly involved in the motor reaction and visuo-spatial task (*3dDeconvolve*), by modeling each type of condition with a separate regressor, obtained by convolution of the task with a standard hemodynamic response model. The six movement parameters derived from the volume registration and the polynomial regressors accounting for baseline shifts and linear/quadratic drifts in each scan series were included in the multiple regression analysis as regressors of no interest.

Individual unthresholded responses for each of the stimuli of interest were transformed into the Talairach and Tournoux Atlas [44] coordinate system, and resampled into 1 mm^3^ voxels for group analyses. Activations were anatomically localized on the naïve and professional group-averaged Talairach-transformed T1-weighted images, and visualized using normalized SUMA surface templates.

We used a mixed-effect meta-analysis approach (*3dMEMA*) for group analysis by using the unthresholded-weights of each condition of interest to construct *T* contrasts and identify both significant pattern of neural response during each task (equivalent to *one*-sample group *t*-tests) and significant differences between the two groups (equivalent to a two-tailed unpaired *t*-tests). The MEMA approach assigns the weight of each subject contribution in the final result based on the precision of the β estimation from each subject, so determining a gain in statistical power for the group effect of interest at most regions, and less spurious isolated voxels in the final results [45]. This method has been demonstrated to be more robust against outliers and more reliable in experiments with a relatively small number of subjects when compared to conventional group analysis approaches [45].

The correction of both the one sample group *t*-maps and the unpaired *t*-contrasts for multiple comparisons across whole brain was made using Monte-Carlo simulations run via *AlphaSim* in AFNI with a voxelwise threshold *p* < 0.005 that resulted in a minimum cluster volume of 561 mm^3^ (cluster connection radius 1.73 mm) for a corrected *p* value < 0.05 at a cluster level.

### Multivariate Autoregressive Analysis

We computed a connectivity analysis based on a multivariate autoregressive model [46-49] to investigate task-related brain networks and identify potential differences between the naïve and professional driver groups. This analysis was carried out using the *1dGC* program included in the AFNI package [49,50]. For each of the two tasks, we identified a set of ‘core’ regions of interest (ROIs) consistently activated in both naïve and professional drivers, using the following procedure: (i) group functional results were thresholded using a conservative statistical threshold (uncorrected voxel-wise *p* < 10^-5^, minimum cluster size of 100 mm^3^) and used to compute a conjunction activation map (logical AND); (ii) an across-group activation index was computed within the identified ‘common regions’ by averaging the coefficients of the two groups on a voxel by voxel basis; (iii) the ‘*maxima*’ function available in AFNI was used to draw 5 mm radius spheres centered on the more robust across-group activation peaks separated by at least 40 mm (this value was chosen arbitrarily to obtain a number of ROIs comprised between 5 and 15). For each subject and ROI, concatenated task-related BOLD timeseries were extracted and used to compute connectivity networks at a single subject level. Applied preprocessing steps included slice timing correction, spatial smoothing for noise reduction, and signal normalization for each segment. The covariates used as input for the model included the six movement parameters, a polynomial function modeling the BOLD drifting effect and the gray-matter signal. A lag order of one TR was chosen, according to the Schwarz information criterion for model selection. Finally, obtained path coefficients (indicative of both strength and direction of the temporal relation between ROIs) and corresponding t-statistics were used in a linear mixed-effect multilevel model to compute a group comparison for each task (*p* < 0.05). 

### BOLD Variability Analysis

The mean squared successive difference (MSSD) [51,52] has been adopted as a measure of temporal variability of the BOLD response. The MSSD is based on differences between successive observations and, for this reason, is more appropriate than the Standard Deviation (SD - based on the difference between single observations and the overall mean) to evaluate temporal variability in experiments with different task conditions [52]. 

Data preprocessing included slice timing and motion correction, spatial normalization and smoothing with a 6 mm Gaussian kernel. In addition, voxel time series were further adjusted by regressing out motion correction parameters, a polynomial function modeling the BOLD drifting effect and white matter (WM) and cerebrospinal fluid (CSF) timeseries [37]. WM and CSF time courses were extracted from two small (1 voxel radius) ROIs respectively located in corpus callosum and ventricles of the ‘common template’ obtained by merging spatially normalized anatomical images of all subjects. To optimize MSSD computation on our system, preprocessed functional data were resampled to obtain 3 mm^3^ voxels. For each individual run, MSSD was computed over the entire preprocessed activation time course using a custom built function in MATLAB (The MathWorks, Inc.).

For each subject, obtained MSSD values were averaged across different runs of the same task and an unpaired t-test was used to look for any potential differences between professional and naïve drivers (significance threshold was set at *corrected p* < 0.05, obtained with a voxelwise threshold of *p* = 0.05 and a minimum cluster volume of 158 voxels). 

## Results

### Behavioral Results

**Figure 2:**
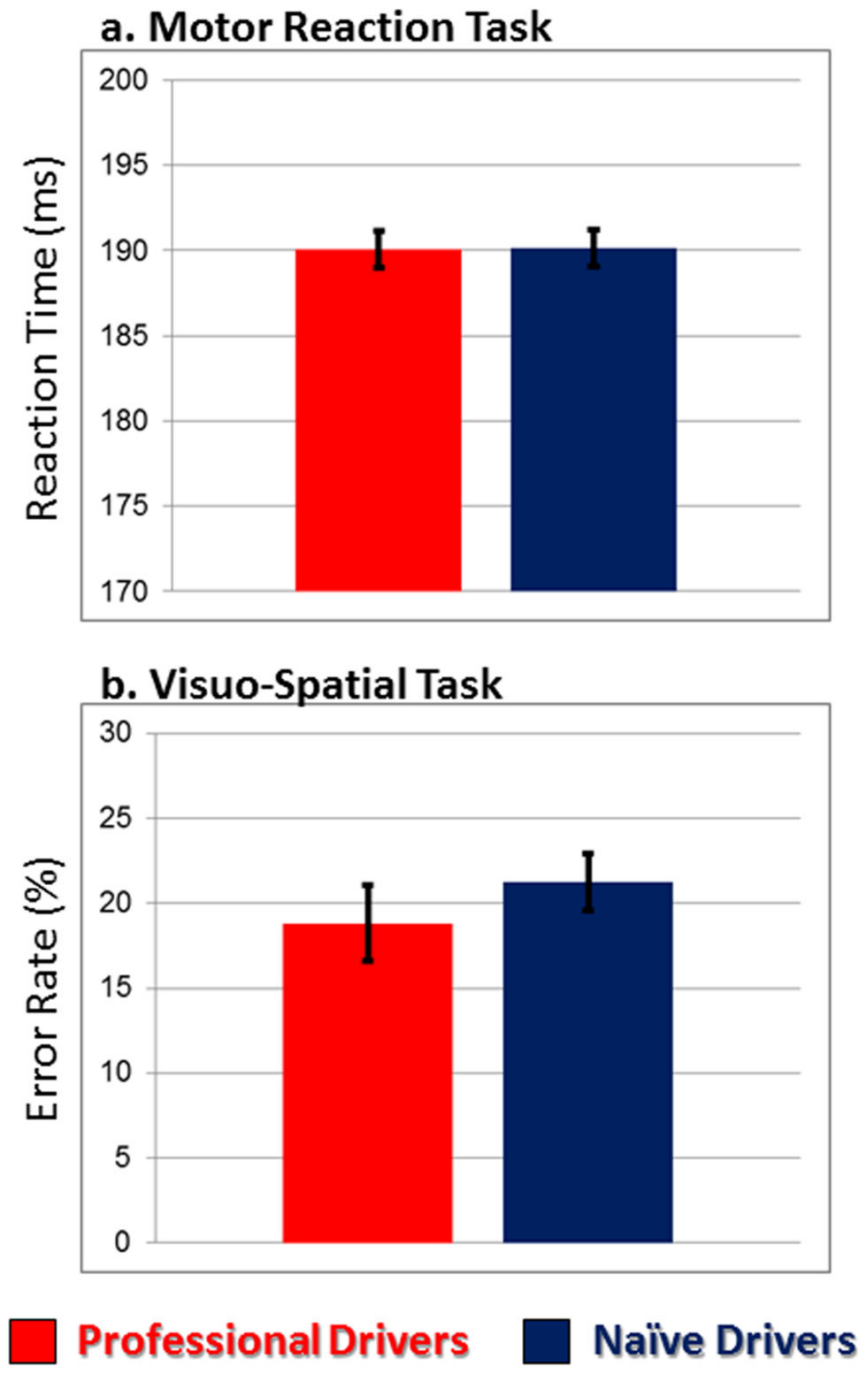
Behavioural results. Behavioural performance of professional (red) and naïve (blue) drivers during the motor reaction task (a) and the visuo-spatial task (b). Average reaction time and mean error rate (± SE) are shown in the graphs. No significant differences between the two groups have been observed.

Professional and naïve car drivers showed no significant differences in performance neither in the motor reaction task (mean reaction time equals to 190.0 ± 28.6 ms and 190.1 ± 32.7 ms, respectively; T_(1,19)_ = -0.08; *p* = n.s.; Figure 2a), nor in the visuo-spatial task (mean error rates were 18.8% ± 9.2 and 21.2% ± 8.2, respectively; T_(1,18)_ = 0.70; *p* = n.s.; Figure 2b), as attended given the experimental task design.

### Task related functional brain responses

**Figure 3:**
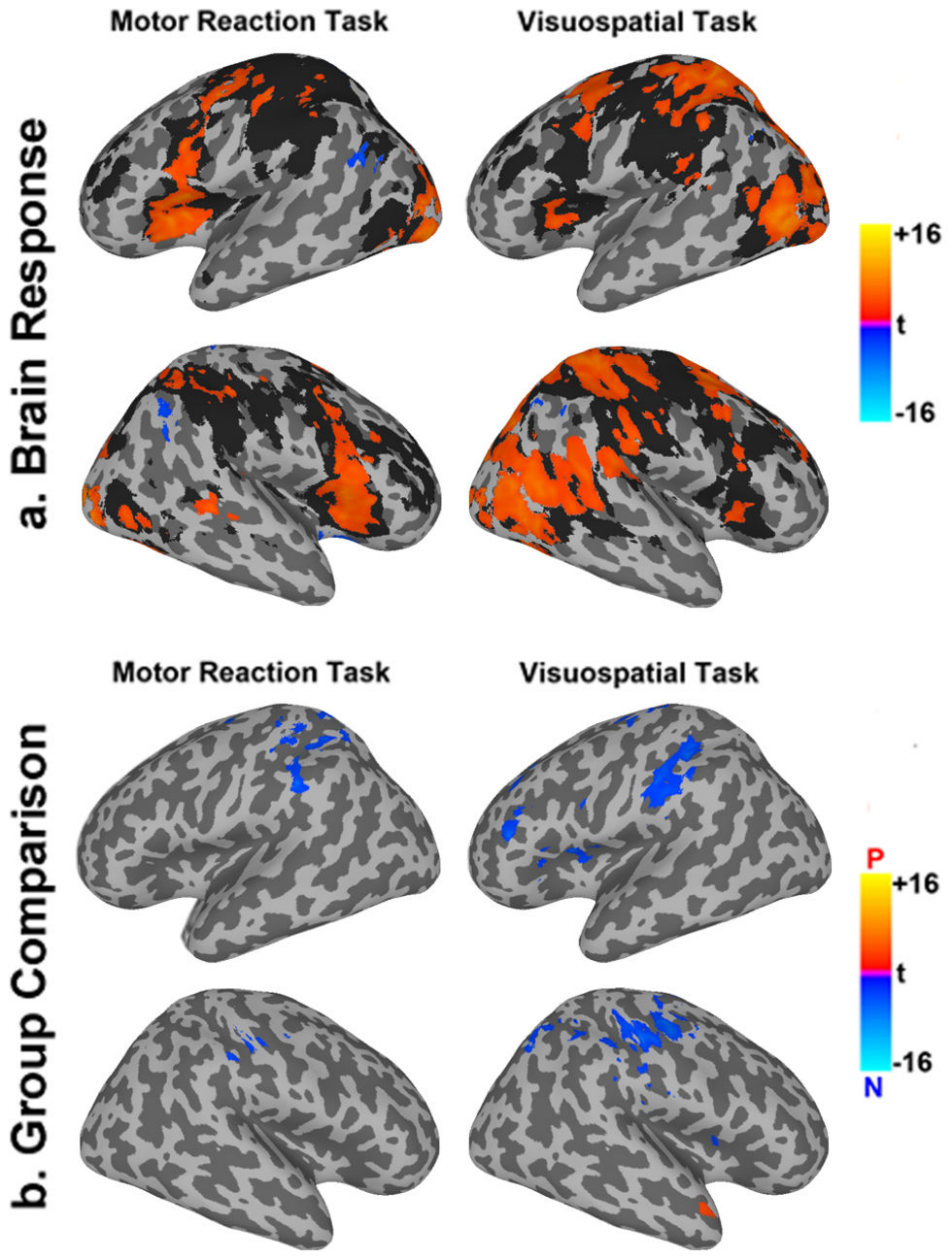
Brain activation results. (a) Brain activations in left (top) and right (bottom) hemispheres of professional (blue/yellow scale) and naive (gray shadow) drivers during motor reaction (1st column) and visuo-spatial (2nd column) tasks computed at whole brain corrected *p* < 0.05. (b) Left (top) and right (bottom) hemispheres activation contrast maps for the two tasks, where blue color corresponds to regions significantly more activated in naive as compared to professional drivers, while red color corresponds to regions significantly more activated in the professional drivers group (*p* < 0.05, whole brain corrected).

During the motor reaction task, both naïve and professional drivers recruited a network of bilateral cerebral regions, including middle and inferior frontal cortex, insula, striatum, cerebellum, cingulate, sensorimotor, temporo-occipital and parietal cortex (Figure 3a). In a mixed-effect analysis, as compared to professional drivers, naïve controls showed a significantly (*p* < 0.05, whole brain corrected) stronger response in right postcentral cortex, left precentral area, left precuneus, left inferior and superior parietal lobules (Figure 3b). 

During the visuo-spatial task, both groups engaged a network of bilateral regions, including middle and inferior frontal cortex, insula, striatum, cerebellum, cingulate, sensorimotor, temporo-occipital and parietal cortex (Figure 3a). Moreover, as compared to professional drivers, naïve subjects showed a significantly (*p* < 0.05, whole brain corrected) greater and more extensive response in supplementary motor area (SMA), left middle frontal and precentral cortex, bilateral inferior parietal lobule, right superior parietal, and postcentral cortex, cerebellum, and bilateral striatum (Figure 3b). Additionally, naïve drivers showed a significantly greater reduction of BOLD response in the right temporopolar area as compared to professional car drivers. 

The Talairach coordinates for the regions that showed a significantly different activation between the two groups during the motor reaction and visuo-spatial task are listed in Table S1.

### Brain functional networks

A multivariate autoregressive analysis was used to search for differences between task-related networks in professional and naïve car drivers. Specifically, we identified two sets of ‘core’ regions of interest (ROIs) on across-group activation peaks: SMA, bilateral insula, bilateral inferior occipital cortex and cerebellum, for the motor reaction task; bilateral dorsal premotor cortex (dPM), bilateral human middle temporal cortex (hMT), right precuneus, left insula, cerebellum and thalamus for the visuo-spatial task. The Talairach coordinates of included ROIs are listed in Table S2.

**Figure 4:**
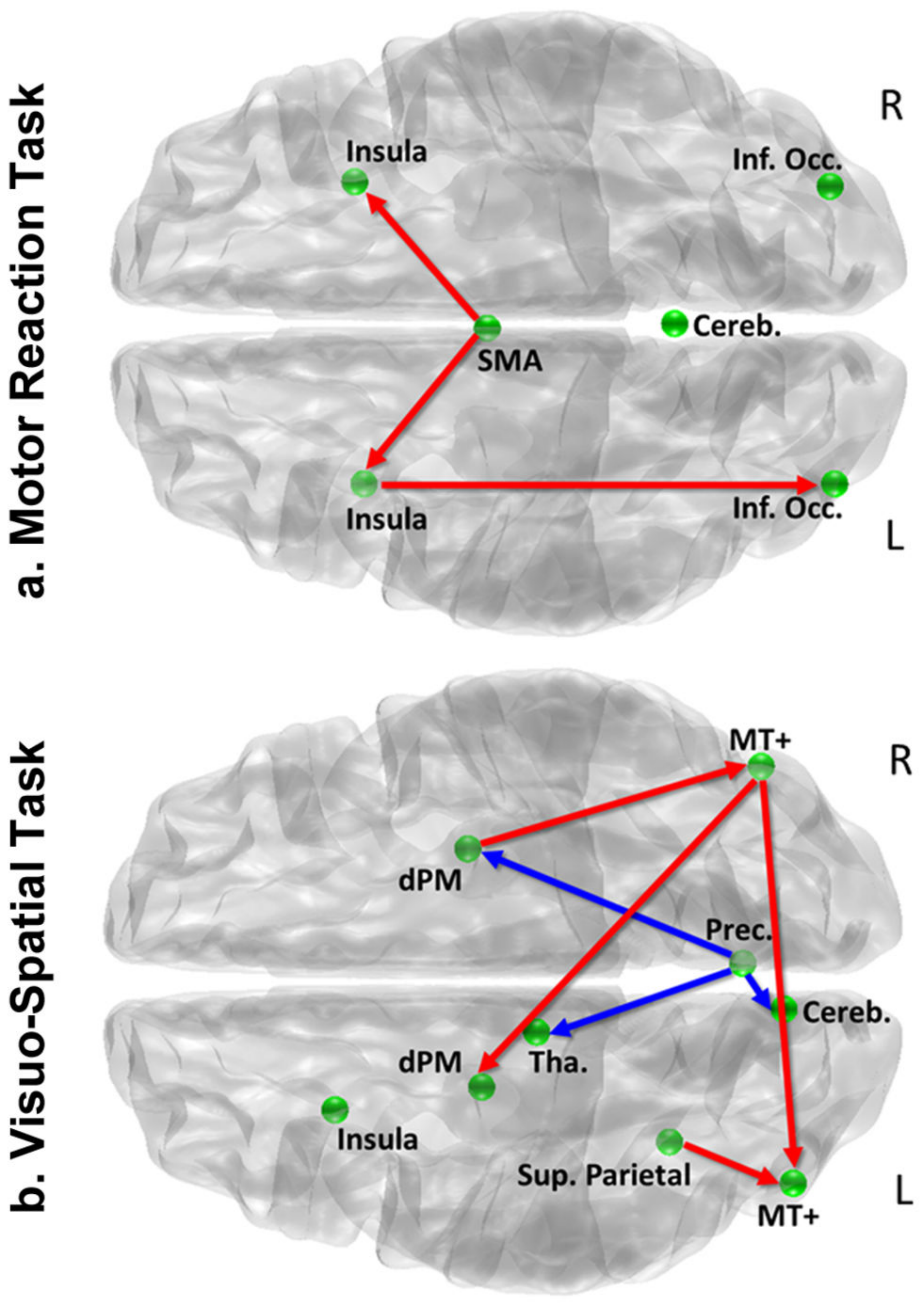
Connectivity Group Comparisons. Statistical map of between group comparisons derived from path coefficients and t-statistics obtained using multivariate autoregressive analysis (MAR) during (a) the motor reaction and (b) the visuo-spatial task. Red and blue arrows respectively indicate significantly greater positive and negative influence on target regions for which a within group effect was also present (*p* < 0.05). Here are shown connections that were significantly stronger in professional as compared to naïve drivers (we observed no stronger connections in this latter group).

During both tasks, the connectivity analysis demonstrated numerous stronger (*p* < 0.05) connections in professional drivers brain networks as compared to naïve drivers (Figure 4). Specifically, during the motor reaction task, activity in SMA predicted subsequent activation in bilateral insula, and activity in right insula predicted activation in right inferior occipital cortex to a significantly greater extent in professional drivers than in the control group (Figure 4a). Similarly, during the visuo-spatial task, professional drivers showed stronger correlations between right hMT+ and left hMT+, right hMT+ and left dPM, left superior parietal cortex and left hMT+, and between right dPM and right hMT+. Moreover, we observed significantly stronger negative correlations between the right precuneus and the right dPM the thalamus and the cerebellum, in professional as compared to naïve drivers (Figure 4b).

The group mean path coefficients observed during the motor reaction and the visuo-spatial task are listed in Table S3 and S4, respectively. 

### BOLD signal temporal variability

**Figure 5:**
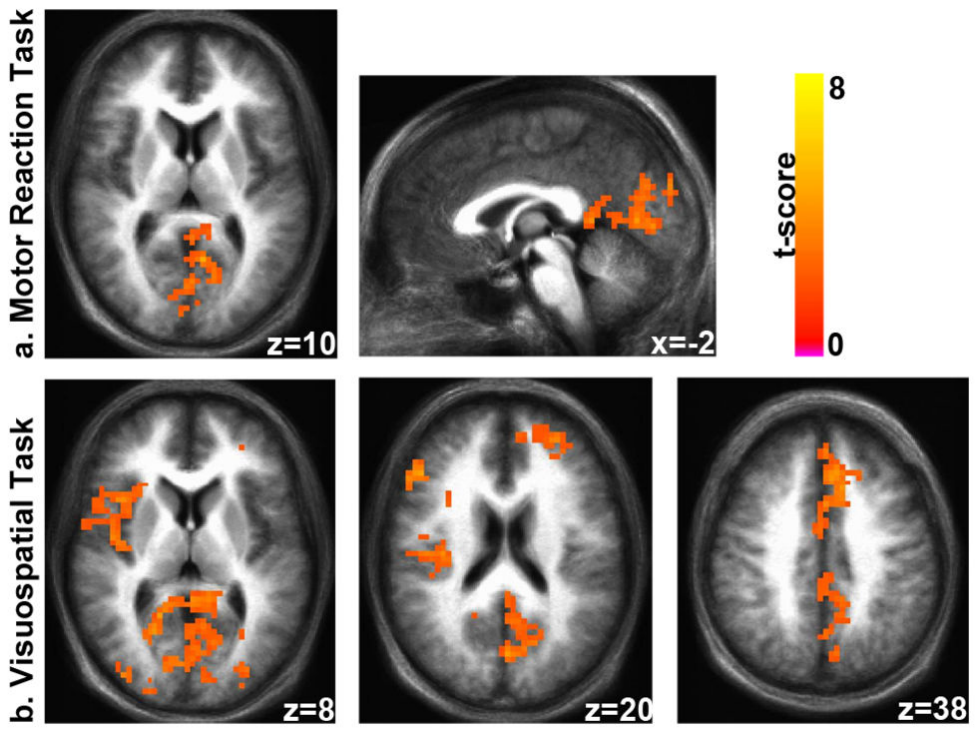
MSSD Group Comparisons. MSSD contrast maps for motor reaction (a) and visuo-spatial (b) task. Yellow/red regions indicate significantly (*p* < 0.05, whole brain corrected) greater BOLD variability in professional as compared to naïve drivers. No regions showed significantly greater MSSD in naïve drivers.

During both the motor reaction and the visuo-spatial tasks, professional, as compared to naïve, drivers showed a significantly higher BOLD signal temporal variability (*p* < 0.05, whole brain corrected) in distinct cortical regions, while no area showed any increased MSSD in the naïve drivers (Figure 5). Specifically, in both tasks, professional drivers showed a greater variability in medial visual areas and posterior cingulate cortex. In addition, in the visuo-spatial task, professional drivers showed differences also in areas including cingulate cortex, bilateral medial and middle frontal areas, right insula and bilateral occipital cortex. 

## Discussion

The present study was designed to investigate the brain functional organization in a sample of top-level professional racing-car drivers, whose brain is required to process visuo-spatial and motor information at a degree that is far beyond the highest level that may arise under physiological situations. In fact, these athletes race at very high speed, have to sustain high concentration levels for extended periods of time, and must rely on fast and accurate visuo-spatial processing and motor response. Therefore, we used fMRI to measure brain functional responses in professional and naïve drivers while they performed simple motor reaction and visuo-spatial tasks. Specifically, we questioned how such high-level skills would affect the brain functional response associated with the execution of ‘everyday’ tasks that did not require any specific training or expertise. To this purpose, the experimental tasks were designed to require basic motor and visuo-spatial abilities available to everyone, in order to avoid any potential effect due to a different performance level between the two groups [42]. Indeed, a significantly different level of performance during more complex tasks would indicate the inability of the lesser skilled subjects to correctly perform the proposed assignment, and therefore would bias any comparison of the neural activity patterns between the two groups. Behavioral results showed that, as expected and desired, performance did not differ on either task between professional and naïve drivers. 

### Reduced activation in task-related cortical areas in skilled drivers

During the motor reaction and the visuo-spatial tasks, both naïve and professional drivers recruited similar distributed networks of bilateral cortical regions, which included areas devoted to visuo-spatial processing (occipital, posterior temporal and parietal cortex), motor control (motor and premotor areas, striatum and cerebellum), and executive functions (middle and inferior frontal cortex). Interestingly, while the observed activation patterns showed a clear spatial overlap across the two groups, in both tasks, professional drivers recruited task-related brain areas, including sensorimotor, parietal, and prefrontal regions, to a significantly smaller extent as compared to naïve subjects. These findings are in agreement with results obtained in other skilled groups, including musicians [53-57], golf players [9,10,12] and pistol shooters [13,58], and indicate an increased efficiency in attentional and sensory information processing along with a reduced ‘resource consumption’ [27,28,59]. However, increased activations in experts as compared to ordinary individuals also has been reported [60-63], and a number of factors have been claimed to play a role in determining these divergent results, including task complexity and specific cognitive task requirements [29,64]. For instance, the greater cortical recruitment during motor imagery or observation of sport-related activities in divers [62] and archers [60], as compared to non-athletes, may be explained by the different confidence of the two groups with the involved motor acts. In fact, it is now clear that a specific motor expertise is required to obtain an actual motor representation in the human brain [65-67]. On the other hand the increased cortical activation in the archers group during a visuo-spatial working memory task [61] could be explained by the fact that this task did not directly reflect the specific skill domain of these athletes [3]. 

### Increased regional correlations during visuo-motor processing

Measures of connectivity were used to investigate whether the described differences in the brain functional response were accompanied by modifications in the way task-related regions intercommunicate. During both the motor reaction and the visuo-spatial task, professional as compared to naïve drivers were characterized by a reinforced connectivity between a number of task-related areas. In particular, during the motor reaction task professional drivers revealed an increased correlation between SMA and bilateral insula, two regions that play a key role in response inhibition and preparation of motor acts [68,69]. Similarly, during the visuo-spatial task, professional drivers showed stronger correlations between a motion detection devoted area, that is, the human MT complex [70], and parietal and premotor cortical regions, suggesting an enhanced information flow during visuo-spatial processing. These results are consistent with previous experiments indicating that superior skills or expertise acquisition are associated with strengthened functional correlations between brain areas involved in sensory processing and motor control [20,21,26,58,71,72]. While also opposite or mixed evidence have been reported, for instance in studies on the functional correlates of cognitive efficiency in working memory [25], a commonly accepted explanation for these results is that a greater neural efficiency may be associated with a reinforcement of the essential task-related connections and a pruning of the superfluous ones [29,73]. As a matter of fact, regions that are not activated during task execution in the skilled group have a lower probability to show a high degree of interaction (and thus, of connectivity measures) with other brain areas. Given these premises, to avoid any potential circularity, here we focused only on brain regions that were strongly activated in both groups during each task. 

Our connectivity results are consistent with the reduced regional activation described above, and altogether indicate that skills and expertise in these professional drivers are accompanied by relevant brain functional modifications. Importantly, such a distinct brain functional organization emerges even during relatively simple visuo-motor tasks that do not require any specific expertise. 

### BOLD signal variability as a marker of neural efficiency

A greater temporal variability of BOLD signal, as measured by using the MSSD statistic, was observed in professional as compared to naïve drivers during both the motor reaction and the visuo-spatial tasks. Recently, BOLD signal variability has been proposed as a novel potential index of ‘brain age’ and ‘operative efficiency’. In fact, younger, better performers exhibit significantly higher signal variability in a number of brain cortical areas when compared to older, poorer-performing individuals [37,38]. Therefore, an increase in temporal variability may indicate a more sophisticated neural system, capable to better adapt to rapid changes in environmental demands, with a more efficient use of cognitive resources [74,75]. Interestingly, the pattern of MSSD differences observed in the present study is similar to the one described in the comparison between young and older individuals [38]. Therefore, our results indicate that brain signal variability may represent a useful and reliable index not only to distinguish different age groups but, from a broader perspective, to individuate more general differences in the quality of brain operative functioning [39,40,76]. 

The present findings complement the information obtained from the comparison of functional brain activity between young and older adults, as well as from the comparison of healthy and cognitively impaired older individuals [35,36,77,78]. In fact, here we explored the ‘opposite direction’ of the spectrum, as we compared ‘common’ and ‘super-skilled’ young adults. In this respect, the brain functional correlates observed in the highly skilled individuals seem to mirror the changes associated with aging. Indeed, as the brain of younger individuals is characterized by a greater neural efficiency as compared to older individuals, the brain of elite athletes, such as professional racing-car drivers, may be considered someway ‘younger’ (thus, more efficient) than the one of age-matched non-athletes.

### Neural efficiency and driving behavior

Although the two tasks adopted in the present study did not comprise any direct performance of (simulated) driving, they required basilar motor and visuo-spatial abilities that are fundamental functions for driving behavior. Indeed, while driving, individuals have to visually monitor the environment around them to identify potential imminent or forthcoming dangers, and have to put in place prompt and accurate motor responses [79]. Consistently, recent functional studies demonstrated that driving a car requires the recruitment of brain regions previously associated with functions such as attention, motor control, visuo-spatial perception and decision making, including striate and extrastriate cortex, superior and inferior parietal lobules, lateral prefrontal cortex and sensori-motor areas [79-82]. As a matter of fact, most of these areas were also recruited during the two tasks adopted in the present study, suggesting the possibility of shared anatomical, functional and cognitive substrates with driving-related activities. Although to our knowledge no studies yet investigated the functional differences between every-day driving and high-speed competitive car-racing, this latter condition is unquestionably more demanding for the human brain, mostly due to the extremely high speeds and the consequent need for a sustained high level of attention and a very efficient visuo-motor processing. Specifically, these highly demanding driving conditions, and the associated mental and physical training activities, may have contributed to the development of the distinctive functional organization observed in the present work in professional car racers during simple motor and visuo-spatial tasks.

Finally, it is worth noting that the concept of neural efficiency, as it is emerging from the existent literature and the present work, may have relevant practical implications for many human activities, including every-day driving. In fact, one can speculate that neural efficiency could favor an increased resistance to cognitive fatigue during long tasks because of a reduced resource consumption (e.g., [83]). In particular, driving is a complex task in which errors can easily emerge at different levels, especially when the speed is high and/or a decrease in attention level is favored by a long and monotonous driving route [82,84]. In this perspective, future studies should investigate a potential relationship between neural efficiency, time-on-task and performance, and the potential benefice of training programs aimed at improving neural efficiency, especially for individuals exposed to longer driving times, such as bus or track drivers.

### Limitations of the study

The number of subjects in this study may appear to be relatively limited in light of the current standards of many fMRI experiments [85]. Nonetheless, it is important to keep in mind the exceptionality of the athletes sample, as the number of professional racing-car drivers with top level careers in Formula One, or similar racing categories (e.g., World Series, Formula 3), is very limited to begin with. Moreover, we obtained consistently significant findings across two independent tasks and three different analysis approaches, thus indicating that the functional changes were statistically robust despite the relatively low number of participants.

The interpretation of results obtained using connectivity analysis based on the measure of time-lagged influence in fMRI also ought to be considered with caution, due to intrinsic technical and physiological limitations of the method (e.g., [86]). Specifically, individual regional variability and the sluggishness of hemodynamic response measured by fMRI may represent potential confounding factors. Although we cannot completely exclude these issues in our case, the fact that we obtained consistent results evaluating different networks recruited during two unrelated visuo-motor tasks, strongly support our interpretation. Moreover, we carried out group comparisons using an approach based on a linear mixed-effect meta-analysis [45,49], which allowed us to use individual information about both effect size and variance, thus accounting for across and within subject variability. Finally, our findings also are supported by previous functional studies indicating that the increased neural efficiency associated with practice and expertise usually is expressed by a reduced brain response extension and a network reorganization, with strengthened connectivity measures between key regions involved in a defined task [3].

## Conclusions

To our knowledge, the present study is the first to provide an integrated and consistent analysis of three indices of brain functional activity associated with intensive training and exposure to extreme conditions in a unique sample of highly skilled athletes, namely professional Formula racing-car drivers. Using two independent visuo-spatial and motor reaction tasks, we showed that in professional racing-car drivers a reduced regional neural response is associated with a reinforced connectivity among task-related cortical areas, as compared to naïve control subjects. In addition, the professional drivers were characterized by an increased BOLD signal variability, a feature previously found in younger individuals as compared to poorer performing older adults. These findings demonstrate that visuo-motor processing in highly-skilled individuals is sustained by a different brain functional architecture, with both ‘quantitative’ and ‘qualitative’ differences in brain recruitment as compared to naïve subjects. Indeed, the brain of highly skilled individuals processes visuo-motor information in a clearly distinctive manner even when subjects are requested to perform relatively simple, non-demanding tasks, in which naïve individuals have equal levels of performance. These results are consistent with and further expand findings from other skilled groups, including musicians, and provide novel evidence to the hypothesis of an increased ‘neural efficiency’ in highly skilled individuals [29,59]. Finally, from a wider perspective, the described results are particularly relevant in light of the recent interest toward the use of physical and mental trainings as ways to contrast or slow down cognitive impairment associated with physiological or pathological aging [87], as they strongly support the potential role of psycho-physical trainings in maintaining a functionally ‘elastic’ and efficient brain. 

## Supporting Information

Table S1 **Talairach coordinates for the centers of mass of voxel clusters that showed significantly different activation in the two groups during the motor reaction task and the visuo-spatial task.**
(DOC)

Table S2 **Talairach coordinates for the centers of mass of regions of interest (ROIs) included in the multivariate autoregressive (MAR) analyses for the motor reaction task and the visuo-spatial task.**
(DOC)

Table S3 **Group mean path coefficients during motor reaction task, with prediction going from row to column.** The group means in bold are significantly different from zero (p<0.05, uncorrected). Inf.Occ., inferior occipital cortex; SMA, supplementary motor area; Cereb., cerebellum.
(DOC)

Table S4 **Group mean path coefficients during visuo-spatial task, with prediction going from row to column.** The group means in bold are significantly different from zero (p<0.05, uncorrected). Prec., precuneus; MT+, middle temporal complex; S.Par., superior parietal cortex; dPM, dorsal premotor cortex; Cereb., cerebellum; Tha., thalamus.
(DOC)
