# Supplementary material for: How Skill Expertise Shapes the Brain Functional Architecture: An fMRI Study of Visuo-Spatial and Motor Processing in Professional Racing-Car and Naïve Drivers
Source: PLoS One. 2013 Oct 18;8(10):e77764. doi: 10.1371/journal.pone.0077764 (PMC3799613; doi:10.1371/journal.pone.0077764)
Supplement: Table S1 — Talairach coordinates for the centers of mass of voxel clusters that showed significantly different activation in the two groups during the motor reaction task and the visuo-spatial task. (DOC) [file pone.0077764.s001.doc]

|  | **Left Hemisphere** | | | **Right Hemisphere** | | |
| --- | --- | --- | --- | --- | --- | --- |
|  | **x** | **y** | **z** | **x** | **y** | **z** |
| **Motor Reaction Task** |  |  |  |  |  |  |
| Inferior Parietal Lobule | -45 | -34 | 43 | - | - | - |
| Postcentral Gyrus | - | - | - | 52 | -25 | 40 |
| Precuneus | -9 | -63 | 58 | - | - | - |
| Precentral Gyrus | -20 | -17 | 56 | - | - | - |
| Posterior Intraparietal Sulcus | -30 | -53 | 55 | - | - | - |
| **Visuo-spatial Task** |  |  |  |  |  |  |
| Cerebellum | -18 | -54 | -12 | - | - | - |
| Middle Frontal Gyrus | -35 | 23 | 20 | - | - | - |
| SMA | - | - | - | 7 | -4 | 47 |
| Precentral Gyrus | -26 | -23 | 58 | - | - | - |
| Postcentral Gyrus | -50 | -24 | 25 | 47 | -24 | 46 |
| Inferior Parietal Lobule | -37 | -34 | 35 | - | - | - |
| Superior Parietal Lobule | - | - | - | 34 | -63 | 44 |
| Superior Temporal Gyrus | - | - | - | 43 | 4 | -15 |
| Striatum | -24 | -10 | 14 | 24 | -7 | 14 |
