# Supplementary material for: How Skill Expertise Shapes the Brain Functional Architecture: An fMRI Study of Visuo-Spatial and Motor Processing in Professional Racing-Car and Naïve Drivers
Source: PLoS One. 2013 Oct 18;8(10):e77764. doi: 10.1371/journal.pone.0077764 (PMC3799613; doi:10.1371/journal.pone.0077764)
Supplement: Table S2 — Talairach coordinates for the centers of mass of regions of interest (ROIs) included in the multivariate autoregressive (MAR) analyses for the motor reaction task and the visuo-spatial task. (DOC) [file pone.0077764.s002.doc]

| **Region of Interest** | **Left Hemisphere** | | | **Right Hemisphere** | | |
| --- | --- | --- | --- | --- | --- | --- |
|  | **x** | **y** | **z** | **x** | **y** | **z** |
| **Motor Reaction Task** |  |  |  |  |  |  |
| Inferior Occipital Cortex | -30 | -82 | -12 | 35 | -83 | -6 |
| Insula | -31 | 20 | 9 | 35 | 18 | 7 |
| SMA | - | - | - | 1 | -6 | 57 |
| Cerebellum | 0 | -49 | -13 | - | - | - |
| **Visuo-spatial Task** |  |  |  |  |  |  |
| Dorsal Premotor Cortex | -23 | -6 | 58 | 29 | -3 | 58 |
| Middle Temporal Complex | -44 | -74 | 6 | 47 | -68 | 6 |
| Superior Parietal Lobule | -35 | -46 | 60 | - | - | - |
| Precuneus | - | - | - | 4 | -61 | 60 |
| Insula | -28 | -23 | 8 | - | - | - |
| Thalamus | -11 | -20 | 16 | - | - | - |
| Cerebellum | -6 | -73 | -12 | - | - | - |
