# Supplementary material for: How Skill Expertise Shapes the Brain Functional Architecture: An fMRI Study of Visuo-Spatial and Motor Processing in Professional Racing-Car and Naïve Drivers
Source: PLoS One. 2013 Oct 18;8(10):e77764. doi: 10.1371/journal.pone.0077764 (PMC3799613; doi:10.1371/journal.pone.0077764)
Supplement: Table S3 — Group mean path coefficients during motor reaction task, with prediction going from row to column. The group means in bold are significantly different from zero (p<0.05, uncorrected). Inf.Occ., inferior occipital cortex; SMA, supplementary motor area; Cereb., cerebellum. (DOC) [file pone.0077764.s003.doc]

| **Prof.** | **L-Inf.Occ.** | **SMA** | **R-Inf.Occ.** | **R-Insula** | **L-Insula** | **Cereb.** |
| --- | --- | --- | --- | --- | --- | --- |
| **L-Inf.Occ.** | -0.018 | -0.026 | **0.093** | -0.053 | -0.030 | 0.014 |
| **SMA** | **0.464** | **0.179** | **0.264** | **0.142** | **0.121** | **0.162** |
| **R-Inf.Occ.** | 0.041 | -0.031 | -0.033 | **-0.057** | -0.042 | 0.014 |
| **R-Insula** | **0.286** | **0.107** | **0.291** | -0.026 | -0.004 | 0.072 |
| **L-Insula** | 0.117 | -0.029 | 0.042 | 0.058 | -0.056 | -0.005 |
| **Cereb.** | -0.018 | 0.010 | 0.029 | -0.040 | -0.031 | **-0.150** |

| **Naïve** | **L-Inf.Occ.** | **SMA** | **R-Inf.Occ.** | **R-Insula** | **L-Insula** | **Cereb.** |
| --- | --- | --- | --- | --- | --- | --- |
| **L-Inf.Occ.** | 0.058 | -0.032 | 0.055 | -0.036 | -0.036 | 0.003 |
| **SMA** | 0.159 | **0.086** | 0.118 | 0.033 | -0.007 | **0.092** |
| **R-Inf.Occ.** | 0.035 | -0.023 | 0.004 | -0.039 | -0.020 | 0.045 |
| **R-Insula** | **0.138** | 0.079 | **0.113** | -0.049 | 0.033 | 0.065 |
| **L-Insula** | 0.113 | 0.087 | 0.105 | **0.090** | 0.007 | 0.049 |
| **Cereb.** | 0.037 | -0.043 | -0.019 | **-0.056** | **-0.052** | -0.085 |

| **[P-N]** | **L-Inf.Occ.** | **SMA** | **R-Inf.Occ.** | **R-Insula** | **L-Insula** | **Cereb.** |
| --- | --- | --- | --- | --- | --- | --- |
| **L-Inf.Occ.** | -0.075 | 0.006 | 0.039 | -0.018 | 0.006 | 0.011 |
| **SMA** | 0.304 | 0.093 | 0.146 | **0.109** | **0.128** | 0.070 |
| **R-Inf.Occ.** | 0.006 | -0.008 | -0.036 | -0.017 | -0.022 | -0.032 |
| **R-Insula** | 0.148 | 0.028 | **0.177** | 0.023 | -0.037 | 0.007 |
| **L-Insula** | 0.004 | -0.116 | -0.063 | -0.031 | -0.064 | -0.054 |
| **Cereb.** | -0.056 | 0.053 | 0.049 | 0.015 | 0.021 | -0.065 |
