# Supplementary material for: How Skill Expertise Shapes the Brain Functional Architecture: An fMRI Study of Visuo-Spatial and Motor Processing in Professional Racing-Car and Naïve Drivers
Source: PLoS One. 2013 Oct 18;8(10):e77764. doi: 10.1371/journal.pone.0077764 (PMC3799613; doi:10.1371/journal.pone.0077764)
Supplement: Table S4 — Group mean path coefficients during visuo-spatial task, with prediction going from row to column. The group means in bold are significantly different from zero (p<0.05, uncorrected). Prec., precuneus; MT+, middle temporal complex; S.Par., superior parietal cortex; dPM, dorsal premotor cortex; Cereb., cerebellum; Tha., thalamus. (DOC) [file pone.0077764.s004.doc]

| **Prof.** | **R-Prec.** | **L-MT+** | **R-MT+** | **L-Sup.Par.** | **R-dPM** | **L-dPM** | **Cereb.** | **L-Insula** | **L-Tha.** |
| --- | --- | --- | --- | --- | --- | --- | --- | --- | --- |
| **R-Prec.** | **-0.157** | **-0.107** | **-0.119** | **-0.090** | **-0.082** | **-0.052** | **-0.129** | **-0.064** | **-0.110** |
| **L-MT+** | **0.220** | 0.024 | **0.086** | **0.066** | **0.085** | **0.068** | 0.055 | 0.016 | 0.011 |
| **R-MT+** | **0.370** | **0.183** | 0.077 | **0.114** | **0.105** | **0.121** | **0.095** | -0.001 | **0.069** |
| **L-S.Par.** | **0.215** | **0.121** | **0.068** | 0.000 | -0.031 | -0.014 | **0.115** | -0.026 | **0.042** |
| **R-dPM** | **0.404** | **0.114** | **0.238** | **0.127** | **0.172** | **0.118** | **0.166** | **0.105** | **0.084** |
| **L-dPM** | **0.331** | **0.185** | **0.139** | **0.185** | **0.132** | 0.026 | **0.166** | 0.075 | 0.057 |
| **Cereb.** | **0.306** | -0.035 | -0.040 | 0.013 | -0.013 | -0.020 | **0.135** | -0.009 | 0.051 |
| **L-Insula** | **0.249** | 0.102 | **0.095** | **0.110** | **0.109** | 0.003 | **0.164** | 0.019 | **0.089** |
| **L-Tha.** | **0.286** | 0.045 | 0.048 | 0.016 | 0.046 | -0.013 | **0.263** | 0.035 | 0.024 |

| **Naïve** | **R-Prec.** | **L-MT+** | **R-MT+** | **L-Sup.Par.** | **R-dPM** | **L-dPM** | **Cereb.** | **L-Insula** | **L-Tha.** |
| --- | --- | --- | --- | --- | --- | --- | --- | --- | --- |
| **R-Prec.** | -0.020 | **-0.080** | **-0.065** | **-0.094** | **-0.046** | **-0.045** | **-0.064** | **-0.055** | **-0.063** |
| **L-MT+** | **0.285** | 0.081 | **0.142** | **0.107** | 0.066 | **0.062** | **0.091** | 0.014 | **0.053** |
| **R-MT+** | **0.378** | 0.070 | 0.074 | **0.086** | 0.030 | 0.036 | 0.042 | -0.022 | 0.018 |
| **L-S.Par.** | 0.049 | 0.013 | -0.003 | 0.054 | 0.003 | 0.025 | 0.065 | 0.027 | 0.017 |
| **R-dPM** | **0.325** | **0.112** | **0.132** | **0.137** | **0.107** | **0.136** | **0.090** | 0.037 | 0.022 |
| **L-dPM** | **0.466** | **0.222** | **0.156** | **0.261** | **0.116** | 0.046 | **0.157** | 0.066 | **0.074** |
| **Cereb.** | **0.224** | -0.048 | -0.027 | -0.017 | -0.008 | -0.033 | 0.049 | -0.005 | -0.013 |
| **L-Insula** | **0.205** | **0.135** | 0.045 | **0.228** | 0.055 | 0.055 | **0.126** | -0.025 | **0.088** |
| **L-Tha.** | **0.514** | 0.098 | 0.084 | **0.109** | 0.023 | **0.078** | **0.196** | 0.015 | -0.015 |

| **[P-N]** | **R-Prec.** | **L-MT+** | **R-MT+** | **L-Sup.Par.** | **R-dPM** | **L-dPM** | **Cereb.** | **L-Insula** | **L-Tha.** |
| --- | --- | --- | --- | --- | --- | --- | --- | --- | --- |
| **R-Prec.** | **-0.137** | -0.027 | -0.055 | 0.004 | **-0.037** | -0.007 | **-0.065** | -0.009 | **-0.048** |
| **L-MT+** | -0.065 | -0.057 | -0.056 | -0.041 | 0.020 | 0.006 | -0.036 | 0.002 | -0.043 |
| **R-MT+** | -0.008 | **0.114** | 0.002 | 0.028 | 0.075 | **0.085** | 0.054 | 0.021 | 0.051 |
| **L-S.Par.** | 0.166 | **0.109** | 0.071 | -0.054 | -0.035 | -0.039 | 0.050 | -0.053 | 0.025 |
| **R-dPM** | 0.079 | 0.002 | **0.106** | -0.011 | 0.065 | -0.018 | 0.076 | 0.068 | 0.062 |
| **L-dPM** | -0.135 | -0.038 | -0.017 | -0.077 | 0.016 | -0.020 | 0.009 | 0.009 | -0.018 |
| **Cereb.** | 0.082 | 0.013 | -0.014 | 0.030 | -0.005 | 0.013 | 0.086 | -0.003 | 0.064 |
| **L-Insula** | 0.043 | -0.033 | 0.049 | -0.118 | 0.054 | -0.052 | 0.037 | 0.044 | 0.001 |
| **L-Tha.** | -0.228 | -0.053 | -0.036 | -0.092 | 0.023 | -0.091 | 0.067 | 0.021 | 0.039 |
